# Multiple Approaches Detect the Presence of Fungi in Human Breastmilk Samples from Healthy Mothers

**DOI:** 10.1038/s41598-017-13270-x

**Published:** 2017-10-12

**Authors:** Alba Boix-Amorós, Cecilia Martinez-Costa, Amparo Querol, Maria Carmen Collado, Alex Mira

**Affiliations:** 1 Institute of Agrochemistry and Food Technology, Spanish National Research Council (IATA-CSIC), Department of Biotechnology, Av. Agustin Escardino 7, 46980 Valencia, Spain; 2 Department of Health and Genomics, Center for Advanced Research in Public Health, FISABIO Foundation, Valencia, Spain; 3 Department of Paediatrics. University of Valencia, Paediatric Gastroenterology and Nutrition Section, Hospital Clínico Universitario de Valencia (Spain), Blasco Ibáñez Av., 17, 46010 Valencia, Spain

## Abstract

Human breastmilk contains a variety of bacteria that are transmitted to the infant and have been suggested to contribute to gut microbiota development and immune maturation. However, the characterization of fungal organisms in milk from healthy mothers is currently unknown although their presence has been reported in the infant gut and also in milk from other mammals. Breastmilk samples from healthy lactating mothers (n = 65) within 1 month after birth were analyzed. Fungal presence was assessed by different techniques, including microscopy, growth and identification of cultured isolates, fungal load estimation by qPCR, and fungal composition using 28S rRNA gene high-throughput sequencing. In addition, milk macronutrients and human somatic cells were quantified by spectrophotometry and cytometry. qPCR data showed that 89% of samples had detectable levels of fungal DNA, at an estimated median load of 3,5 × 10^5^ cells/ml, potentially including both viable and non-viable fungi. Using different culture media, 33 strains were isolated and identified, confirming the presence of viable fungal species. Pyrosequencing results showed that the most common genera were *Malassezia* (44%), followed by *Candida* (19%) and *Saccharomyces* (12%). Yeast cells were observed by fluorescence microscopy. Future work should study the origin of these fungi and their potential contribution to infant health.

## Introduction

Microbiome development in the newborn is a stepwise and crucial process, contributing at the physiological level and influencing the development and maturation of the immune system^1,2^. During delivery, the neonate is exposed to maternal microbes, first from the mother’s reproductive system, rapidly after from the maternal skin and the environment, and later influenced by diet, including breastfeeding. Breastmilk plays an important role in the microbial supply as it contains a variety of potential beneficial bacteria, as well as a wide source of nutrients and essential protective substances that makes it the optimal nutrition for the infant^1,2^. Those bacteria residing in breastmilk are transmitted to the infant during breastfeeding, getting to the intestine and contributing to the settlement of the gut microbiota and acquired immunity^3^. Although bacteria in human milk have been widely assessed, information about the natural presence of fungal species is generally lacking, and it is limited to a few studies focused on mammary infections describing breast candidiasis^4,5^ and a recent metagenomic study on human breastmilk from mothers suffering from mastitis, which confirmed the presence of fungal sequences, in addition to the dominant bacterial fraction^6^. However, fungal presence in the milk of other mammals has been widely described in several studies^7–12^, which supports the idea that human breastmilk could also contain fungi under normal, healthy conditions.

Furthermore, there is evidence that fungal species (yeast-like mainly) can be found in the infant gut early in life^13–17^. The importance of the fungal component -mycobiome- in the human gut has received increased attention by researchers, as it is part of human microbial homeostasis, and changes on it can have direct effects on the host health status^18–20^. It is therefore plausible that colonization of fungal species in the newborn would be important in the early settlement of human microbiota and for immune system development. Therefore, there exists the possibility that breastmilk could be playing an important role in the supply of fungal, as well as bacterial, species to the newborn.

In this pilot study, we aimed to study and identify the presence of fungal species in breastmilk samples from healthy mothers by using molecular approaches and high throughput sequencing, as well as through classical culture methods. We also studied the potential relationships between fungal load and diversity with milk macronutrients composition and human cells’ counts.

## Results

### Viable Fungi in Breastmilk and Fluorescence Microscopy

**Figure 1:**
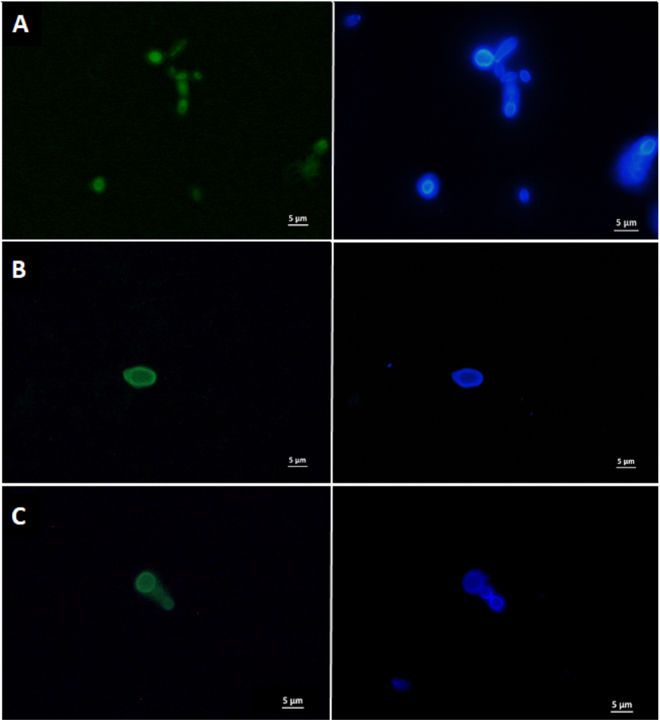
Fluorescent microscopy images of yeasts detected in breastmilk. Left panels are showing the yeasts stained in green with the EUK516 FISH probe targeting the 18S rRNA gene. Right panels are showing the yeasts stained in blue with calcofluor. (**A**) *Candida parapsilosis* isolate FBMI4 (positive control). (**B**) Yeast from fixed transitional breastmilk sample BMF9. (**C**) Yeasts from fixed colostrum sample BMF5.

The cultivable fraction of breastmilk fungi from 41 healthy mothers was investigated through isolation in selective culture media. Fungi were detected in 17 of the samples (representing 41% of all analyzed samples) leading to the identification of 33 isolates, either with one or two primer pairs used in an identification PCR (Table 1). Twenty-five of them were well-assigned to a specific species with one pair of primers or both of them, three presented inconsistencies in the results between the two primers used, and the rest were assigned to uncultured species. The majority of the well-assigned isolates corresponded to the yeasts *Candida parapsilosis* and *Rhodotorula mucilaginosa*, and were found to belong to different strains as inferred from phylogenetic trees (Supplementary Figure 1), discarding contamination from a given strain in the laboratory and supporting the unique presence of both of these species in breastmilk. The presence of fungal species in breastmilk was also confirmed by Fluorescent *in situ* Hybdirization (FISH) targeting the 18 S rRNA gene (EUK516-FITC probe) and by Calcofluor White staining by fluorescence microscopy. Only yeast cells and no hyphal forms were visualized in the analyzed samples (Fig. 1).

**Table 1:** Fungal species isolated in selective growth media.

Isolate Code	Species	Max score^a^	query cover^b^	BLAST e-value	Max identity^c^	Accession N°	Culture medium
FBMI1	*Candida parapsilosis* ^1^	337	94%	6,00E-89	100%	KT876525.1	SB
FBMI2	*Candida parapsilosis* ^1^	324	94%	5,00E-85	99%	KT694025.1	SB
FBMI3	*Uncultured fungus* ^1^	204	97%	4,00E-49	96%	FJ235880.1	SB
FBMI4	*Candida parapsilosis* ^1^	549	98%	2,00E-152	94%	KJ880928.1	SB
FBMI5	*Talaromyces stollii* ^1^	392	61%	3,00E-105	99%	AB910938.1	M
	*Talaromyces purpurogenus* ^2^	568	100%	4,00E-158	99%	KC009578.1	M
FBMI6	*Rhodotorula mucilaginosa* ^1^	350	96%	9,00E-93	99%	EU781664.1	M
	*Rhodotorula mucilaginosa* ^2^	351	59%	7,00E-93	94%	LT220852.1	M
FBMI7	*Candida parapsilosis* ^1^	348	99%	3,00E-92	100%	KU739407.1	M
	*Candida parapsilosis* ^2^	571	99%	3,00E-159	100%	HE605209.1	M
FBMI8	*Rhodotorula mucilaginosa* ^1^	346	100%	1,00E-91	100%	KU724343.1	M
	*Rhodotorula mucilaginosa* ^2^	560	99%	6,00E-156	100%	FJ538169.1	M
FBMI9	*Rhodotorula mucilaginosa* ^1^	335	95%	2,00E-88	98%	KT876599.1	SB
	*Rhodotorula mucilaginosa* ^2^	560	99%	6,00E-156	100%	FJ538169.1	SB
FBMI10	*Cryptococcus diffluens* ^1^	357	99%	5,00E-95	99%	LN808927.1	SB
	Uncultured fungus^2^	569	99%	1,00E-158	100%	KC670786.1	SB
FBMI11	*Rhodotorula mucilaginosa* ^1^	357	100%	5,00E-95	99%	EU781664.1	RB
FBMI12	*Rhodotorula mucilaginosa* ^1^	351	98%	2,00E-93	100%	EU781664.1	RB
FBMI13	Uncultured fungus^2^	58.4	94%	1,00E-04	100%	HQ190233.1	M
FBMI14	*Yarrowia lipolytica* ^2^	488	99%	3,00E-134	99%	AF156969.1	SB
FBMI15	*Yarrowia deformans* ^1^	189	96%	9,00E-45	100%	KY105960.1	RB
	*Yarrowia lipolytica* ^2^	488	99%	3,00E-134	99%	AF156969.1	RB
FBMI16	*Rhodotorula mucilaginosa* ^2^	562	97%	2,00E-156	99%	FJ538169.1	M
FBMI17	*Rhodotorula mucilaginosa* ^1^	316	99%	7,00E-83	100%	KY104848.1	SB
	*Rhodotorula mucilaginosa* ^2^	538	100%	3,00E-149	100%	JQ838010.1	SB
FBMI18	*Saccharomyces cerevisiae* ^2^	564	91%	6,00E-157	99%	KX270743.1	SB
FBMI19	*Rhodotorula mucilaginosa* ^1^	287	90%	6,00E-74	98%	KX866274.1	M
	*Rhodotorula mucilaginosa* ^2^	534	98%	4,00E-148	100%	JQ838010.1	M
FBMI20	Uncultured fungus^1^	250	100%	8,00E-63	92%	FM875845.1	M
	*Rhodotorula mucilaginosa* ^2^	542	100%	2,00E-150	100%	JQ838010.1	M
FBMI21	*Cryptococcus sp*.^1^	281	100%	2,00E-72	100%	KU961663.1	M
FBMI22	*Rhodotorula mucilaginosa* ^1^	315	98%	3,00E-82	100%	KY104848.1	M
	*Rhodotorula mucilaginosa* ^2^	529	100%	2,00E-146	99%	KM222229.1	M
FBMI23	*Rhodotorula mucilaginosa* ^2^	534	99%	3,00E-148	100%	JQ838010.1	SB
FBMI24	Uncultured eukaryote^2^	518	96%	4,00E-143	99%	KT752764.1	RB
FBMI25	*Clavispora lusitaniae* ^1^	196	93%	6,00E-47	99%	KY102561.1	SB
	*Clavispora lusitaniae* ^2^	523	100%	8,00E-145	99%	KP317754.1	SB
FBMI26	*Candida parapsilosis* ^1^	268	98%	2E-68	100%	KY619304.1	M
	*Candida parapsilosis* ^*n*^	549	100%	1E-152	100%	KT199380.1	M
FBMI27	*Candida parapsilosis* ^1^	267	100%	7E-68	99%	KY619304.1	M
	*Candida parapsilosis* ^*n*^	536	98%	1E-148	99%	KT199380.1	M
FBMI28	*Candida parapsilosis* ^1^	333	100%	8E-88	100%	KJ880926.1	YPD
	*Candida parapsilosis* ^2^	531	100%	5E-147	99%	KT199380.1	YPD
FBMI29	*Candida parapsilosis* ^1^	335	97%	2E-88	100%	KT694025.1	SB
	*Candida parapsilosis* ^2^	540	100%	8E-150	100%	KT199380.1	SB
FBMI30	*Candida orthopsilosis* ^2^	510	99%	6E-141	99%	KU058170.1	YPD
FBMI31	*Candida albicans* ^2^	520	100%	1E-143	100%	XR002086442.1	YPD
FBMI32	*Candida albicans* ^2^	438	74%	3E-119	100%	XR002086442.1	YPD
FBMI33	Uncultured Ascomycota^2^	556	93%	9,00E-155	99%	GQ404735.1	YPD

^1^Isolates identified with ITS1-ITS2 primers.

^2^Isolates identified with 18S primers.

^a^BLAST alignment score from the top hit against the NCBI database.

^b^Percentage of query sequence covered by the alignment.

^c^Highest percent identity of all query-subject alignments.

^SB^Sabouraud.

^M^Malassezia CHROMagar.

^RB^Rose Bengal.

### Isolates Phenotypic Characterization

One sample from each of the two most prevalent isolates, *Rhodotorula mucilaginosa* (isolate FBMI6) and *Candida parapsilosis* (isolate FBMI7) were further characterized in order to determine their potential adaptation to human milk. For reference, we also included the *Saccharomyces cerevisiae* isolate (FBMI18), due to its interest for the biotechnological industry.

#### Sugars Utilization and Growth at Different Temperatures

Yeasts growth at different temperatures (28 °C, 37 °C and 42 °C), and their ability to metabolize sugars naturally present in human milk (lactose) are shown in Supplementary Figure 2(a).

The optimal growth temperature for *Saccharomyces* species is usually ~24–26 °C, for *Rhodotorula mucilaginosa* ~24 °C, and for *Candida parapsilosis* ~30 °C-35 °C (temperature conditions according to recommendations from the American Type Culture Collection, www.atcc.org). The fact that we were able to obtain viable isolates of these strains from human milk, which is at a temperature of ~37 °C, suggests an adaptation to the human body. We tested the strains viability at 37 °C and 42 °C. The three isolates were able to grow at any temperature, although the growth of isolate FBMI6 (*R*. *mucilaginosa*) was reduced at 42 °C compared to the other species. The *Sacharomyces cerevisiae* T73 wine strain included as a control was not able to grow at 37 or 42 °C, as expected. The *Saccharomyces cerevisiae* “Cinta Roja” baker strain, adapted to survive high temperatures was also included as a control, and was able to grow at both tested temperatures.

All strains were able to use glucose and sucrose with no growth differences, but none of them were able to grow in the sole presence of lactose, the main sugar present in human milk. Negative control medium (without any sugar) did not support growth of any of the strains.

#### Resistance to Oxidative Stress

The three tested isolates were able to grow in the presence of high levels of hydrogen peroxide H_2_O_2_ (6 mM), and did not differ dramatically from control strains (Supplementary Figure 2(b)).

### Genetic Characterisation of Breastmilk S. cerevisiae Strain

The mtDNA restriction patterns of the isolated *S*. *cerevisiae* strain (FBMI18) from our milk samples was identical to the commercial baker strain “Cinta roja” profile, and the δ-PCR amplification patterns were highly similar, suggesting a correspondence between both strains (Supplementary Figure 2(c)).

### Fungal Load in Breastmilk

**Figure 2:**
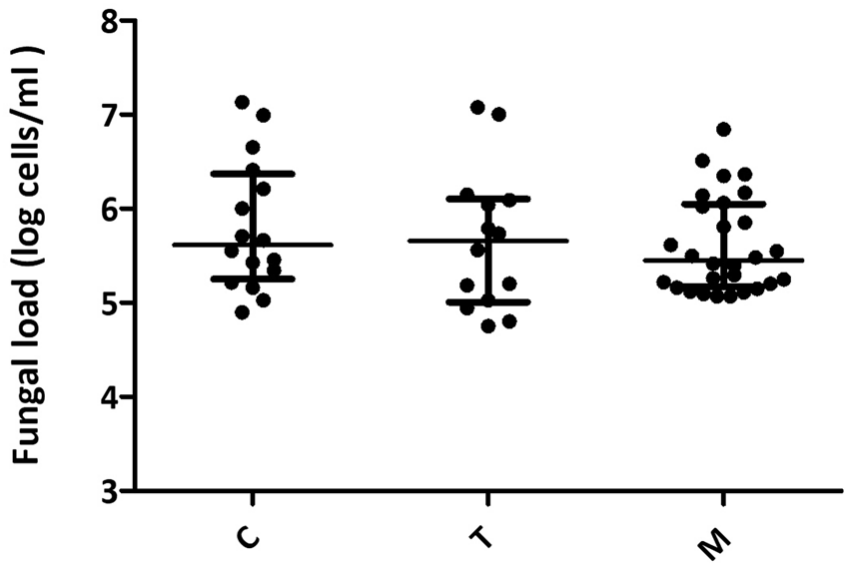
Fungal load in breastmilk over time. The plot shows the median with interquartile ranges of fungal load at three time points in the 89% of samples that showed fungal presence by qPCR. C, colostrum samples (n = 16); T, transitional milk samples (n = 14); M, mature milk samples (n = 28). Detection limit was established at 10^3^ cells/ml, estimated as the lowest concentration at which 95% of the positive samples are detected.

After analyzing 65 milk samples by qPCR, results showed that 58 (89%) had detectable levels of fungi when using primers targeting the ITS1–5.8 S rRNA region, with high inter-individual variability and a total median of 3,5 × 10^5^ cells/ml. Samples were classified in three groups according to their collection time point: colostrum (1st-6th day postpartum), transitional (7th–14th day postpartum) and mature milk (from 15th day onwards). Similar fungal load values at the three time points were observed, with higher levels in colostrum (4,1 × 10^5^ cells/ml) and transitional milk (4,5 × 10^5^ cells/ml) and slightly lower values in mature milk (2,8 × 10^5^ cells/ml) although no statistical differences were found between them (Fig. 2). The prevalence of samples with detectable fungal presence was 16/18 in colostrum, 14/18 in transitional milk and 28/29 in mature milk. Total bacterial load for the same samples was 8,9 × 10^5^ cells/ml when using primers against the single copy gene fusA.

### Fungal Composition in Breastmilk

**Figure 3:**
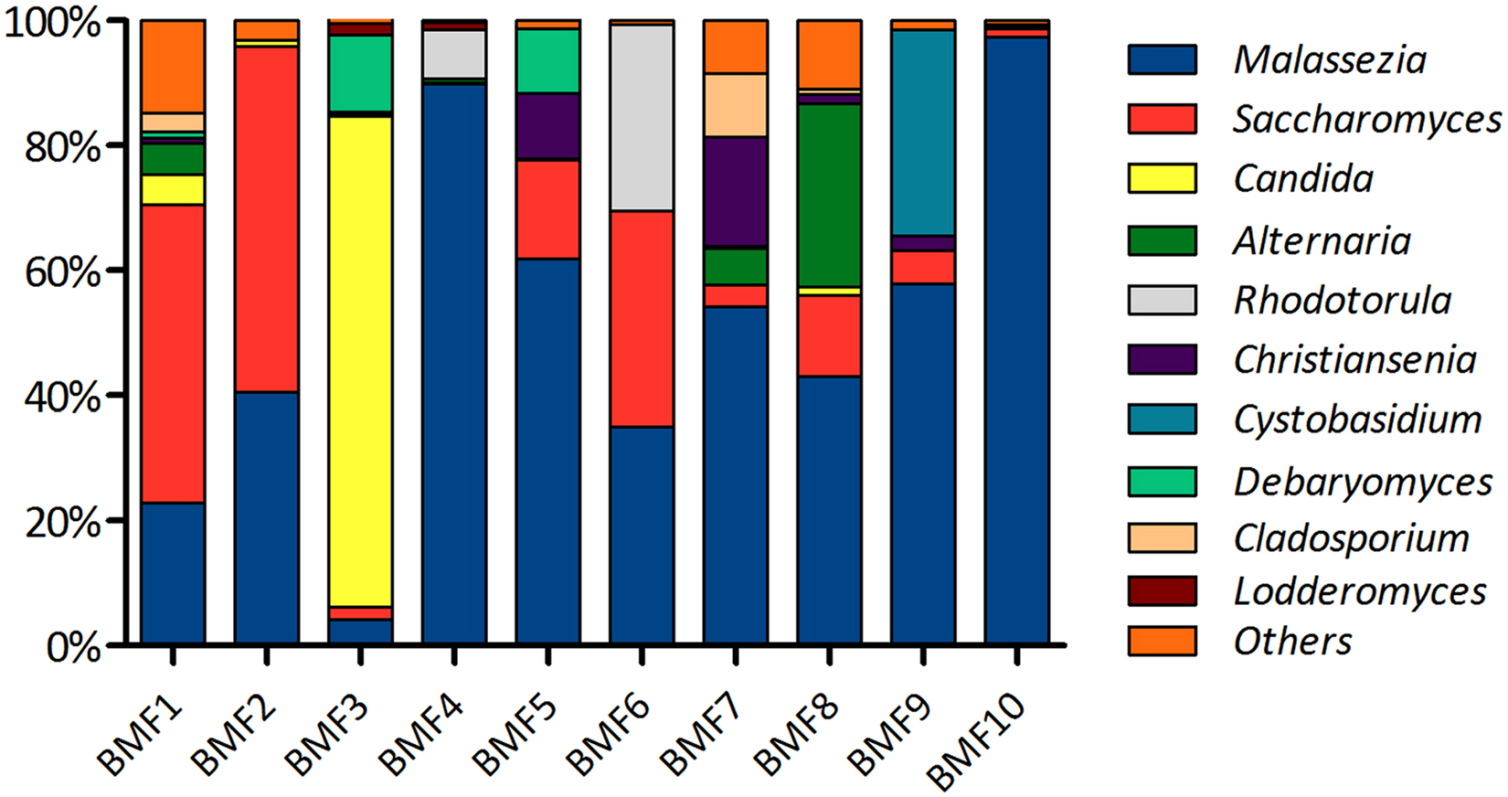
Fungal taxonomic composition of human breastmilk. Bars show the proportion of fungal genera as inferred by PCR amplification and pyrosequencing of the 28S rRNA gene in healthy mothers (n = 10). Each code in the X axis corresponded to a donor. Fungal genera that were under 1% were grouped in the “Others” category. The majority of the samples presented correspond to mature milk samples, except for BMF5 and BMF8 (colostrum) and BMF9 (transitional milk).

To better characterize the breastmilk fungal community of healthy donors, we further analyzed a subset of 15 samples (qPCR-positive for fungi) by means of 28S rRNA gene pyrosequencing using the 454 Roche platform. This technology allows the analysis of long-sequences for reliable taxonomic assignment and prevents the sequencing bias of PCR fragments of variable length such us those of the ITS region. The pyrosequencing of negative controls yielded no sequences. Five of the samples were removed from the analysis as the number of quality-filtered sequences was under 400. We obtained an average number of taxonomically assigned, high-quality sequences of 1,250 per sample (SE: ±444.94), with an average of 580 bp length. 61% of the reads corresponded to the *Basidiomycota* phylum, and 39% to the *Ascomycota* phylum. The analysis led to the identification of 10 classified fungal taxa (to the genus level) which were present in >1% of the total sequences in more than 20% of the samples (Fig. 3). The taxonomic assignment of the sequences showed that the fungal composition of human breastmilk was dominated by *Malassezia*, which corresponded to 44% of the total number of sequences obtained, followed by *Candida* (19%) and *Saccharomyces* (12%). *Malassezia* and *Saccharomyces* could be detected in all 10 samples (Fig. 3).

Through BLAST alignment of the sequences against the RDP LSU database, 30 fungal species could be identified, which corresponded to 9,910 sequences taxonomically assigned after strict filtering (>98% identity and >400 bp length). These species are presented in Table 2 together with their mean percentage and prevalence in the samples.

**Table 2:** Fungal species detected by pyrosequencing, mean proportions per total number of sequences and prevalence. All sequences included in the table had >98% sequence identity over >400 bp alignment length.

Species	%	Prevalence
*Malassezia globosa*	34.63	6/10
*Candida sp*. *HA1671*	19.54	3/10
*Saccharomyces cerevisiae*	14.56	7/10
*Malassezia restricta*	9.07	10/10
*Alternaria arborescens*	4.82	4/10
*uncultured Candida*	3.48	2/10
*Cladosporium bruhnei*	3.28	1/10
*Alternaria sp*. *NT-2015a*	2.67	5/10
*Candida sake*	1.93	2/10
*Alternaria tenuissima*	1.57	5/10
*Debaryomyces hansenii*	1.24	3/10
*Cystobasidium sp*. *CBS7295*	1.20	1/10
*uncultured Debaryomyces*	0.63	3/10
*Candida zeylanoides*	0.33	2/10
*Candida parapsilosis*	0.24	2/10
*Cladosporium sp*. *PAB-2014*	0.22	2/10
*Malassezia sp*.*PH-2014*	0.16	4/10
*Cladosporium delicatulum*	0.12	1/10
*Cladosporium phyllophilum*	0.08	1/10
*Cladosporium cladosporioides*	0.07	1/10
*Cladosporium herbarum*	0.05	2/10
*Alternaria alternata*	0.03	1/10
*Alternaria obclavata*	0.02	1/10
*Alternaria terricola*	0.02	1/10
*Candida albicans*	0.01	1/10
*Cladosporium langeronii*	0.01	1/12

### Relationship Between Breastmilk Microbiota and Milk Components

**Figure 4:**
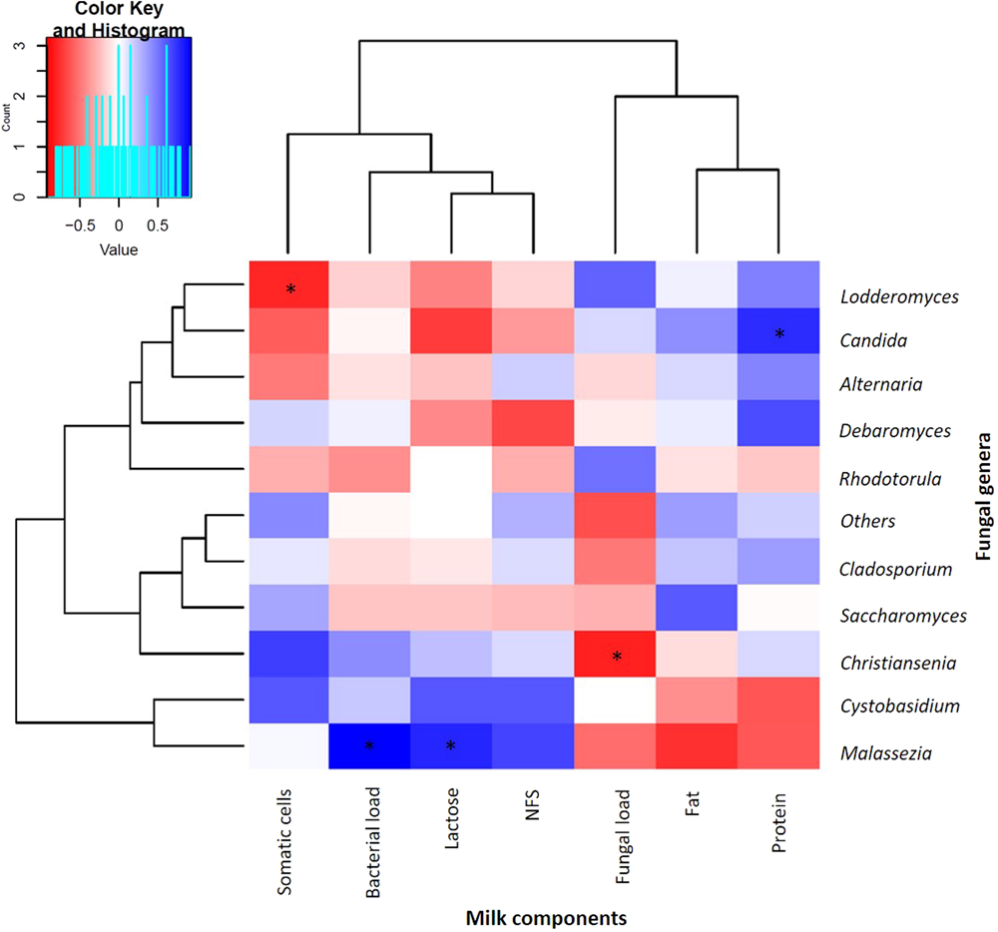
Relationships between fungal taxa relative abundance and nutritional, cellular and bacterial content of human breastmilk. The heatmap shows samples clustered by their compositional profile. Relative abundance of fungal genera is colour-coded according to their negative- (red) or positive- (blue) correlations with the amounts of milk components: fat protein, bacterial load, lactose, fungal load, somatic cells and non-fatty solids (NFS). Significant correlations are represented with an asterisk (*) and are as defined in the results section (n = 10).

Relative abundances of fungal genera from the 10 sequenced milk samples were compared with the amounts of nutritional components (fat, protein, lactose and non-fatty solids), and the number of human somatic cells, as well as with the microbial (bacterial and fungal) load content in paired milk samples, in order to find potential correlations among them (Fig. 4). Positive significant correlations were found between the genus *Malassezia* and bacterial load (Spearman’s correlation coefficient ρ: 0.93, p-value = 0.007); *Malassezia* and lactose (Spearman’s correlation coefficient ρ: 0.78, p-value = 0.048); and *Candida* with protein content (Spearman’s correlation coefficient ρ: 0.77, p-value = 0.044). *Lodderomyces* and human somatic cells showed a negative correlation (Spearman’s correlation coefficient ρ: −0.79, p-value = 0.035), as well as *Christiansenia* and fungal load (Spearman’s correlation coefficient ρ: −0.81, p-value = 0.027).

**Figure 5:**
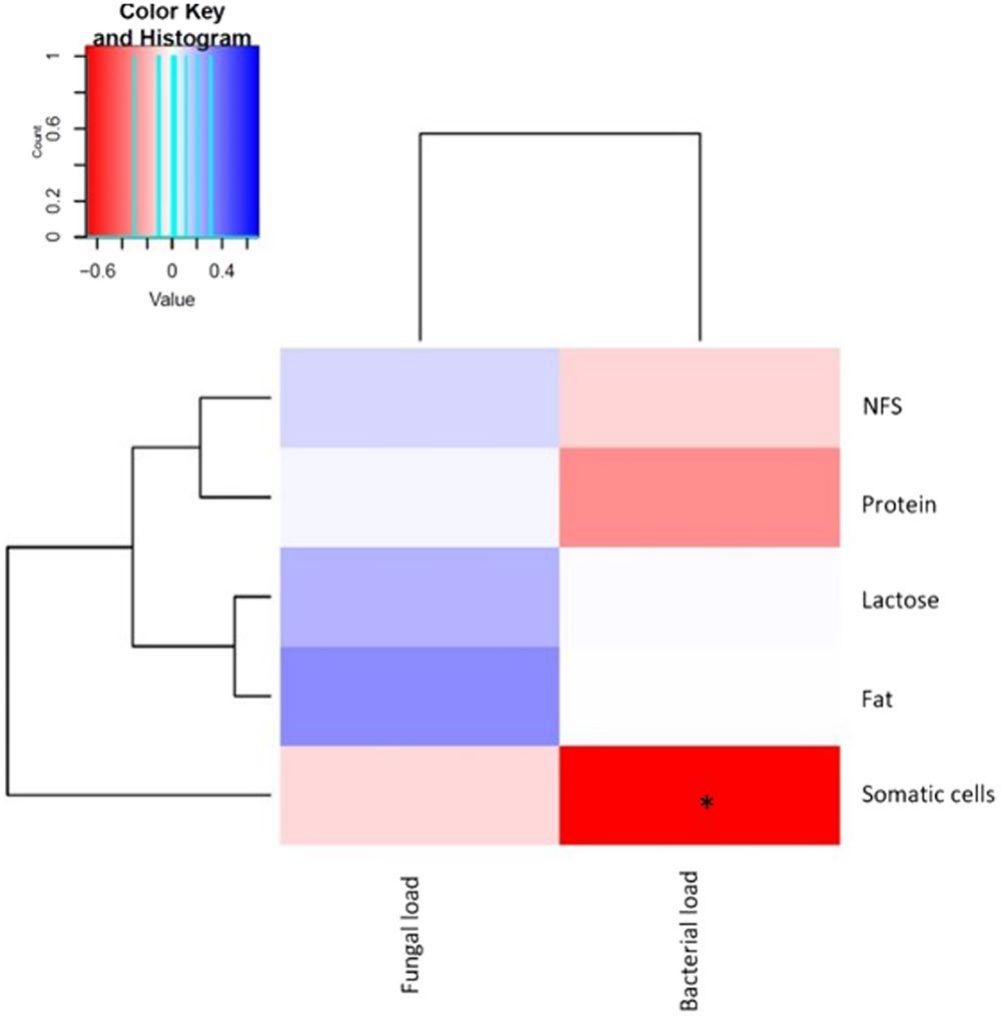
Relationships between fungal and bacterial loads with nutritional and cellular content of human breastmilk. Correlations of fungal and bacterial load appear colour-coded according to their negative- (red) or positive- (blue) correlations with fat content, lactose content, protein content, somatic cells density and non-fatty solids content (NFS). Significant correlations are represented with an asterisk (n = 34).

We also compared nutritional factor composition and human somatic cell numbers with fungal and bacterial load in all of the available samples (n = 34, Fig. 5), and a strong negative correlation was found between bacterial load and human somatic cells (Spearman’s correlation coefficient ρ: −0.69, p-value = 6,28 × 10^–6^). In addition, a non-significant negative correlation was detected between bacterial and fungal load (Spearman’s correlation coefficient ρ: −0.055, p-value: 0.756).

## Discussion

Previous studies have shown that breastmilk harbors an important diversity of bacteria, which are transmitted to the newborn together with many other nutrients and immunological compounds. Those bacteria may have a protective role, activating the immune system and seeding some of the first colonizers in the infant^3^.

Prior to this study, the identification of fungi in milk has only been reported for dairy animals. In the study carried out by Delavenne *et al*., fungal composition from cow’s, ewe’s and goat’s milk were compared, identifying 27 fungal species belonging to a variety of genera, including: *Candida*, *Cryptococcus*, *Debaryomyces*, *Geotrichum*, *Kluyveromyces*, *Malassezia*, *Pichia*, *Rhodotorula*, *Trichosporon*, *Aspergillus*, *Chrysosporium*, *Cladosporium*, *Engyodontium*, *Fusarium*, *Penicillium* and *Torrubiella* ^11^. Spanamberg *et al*. also confirmed the presence of fungi in ewe’s milk^12^.

The possibility that human breastmilk also harbors fungi has not previously been explored. In this work, we have been able to detect a variety of fungal species in breastmilk samples from healthy lactating mothers through different approaches. Fungal culture isolation results revealed that 41% of the samples showed presence of viable fungi. On the other hand, results yielded by qPCR showed that 89% of the samples had detectable levels of fungal DNA, which was present at high proportions with a median load of 3.5 × 10^5^ cells/ml. Bacterial load median for the same samples was 8.9 × 10^5^ cells/ml. This value was close to the results found in a previous study, where the same qPCR protocol was followed to estimate bacterial loads in breastmilk samples from healthy mothers. In this previous study, milk samples were found to have around 10^6^ bacterial cells/ml when detecting the single copy bacterial gene fusA^21^, that is ten-fold higher than the fungal fraction estimated in the current work. However, it should be noted that this fungal estimate is based on the study of the 28S rRNA gene, which is present in a variable number of copies per species, and although we used standard curves from a mix of different fungal species, these measures could overestimate fungal levels, and can’t be compared to results obtained with a single copy gene. No correlation was found between bacterial and fungal load, indicating that the same nutrients or selective pressures that favor a high bacterial density may not be the same that support fungal presence. Nevertheless, a significant correlation was found between bacterial load and the relative abundance of *Malassezia*, and future work should determine if this is the outcome of symbiotic or synergistic relationships as it has been shown in other human samples^22,23^.

In our pilot study, long fragment sequencing of the 28S rRNA gene performed in 15 of the samples allowed reliable taxonomic assignment of the sequences from 10 of them, and showed that breastmilk hosts a variety of fungal genera, with *Malassezia*, *Candida* and *Saccharomyces* being the most abundant. Although their origin is unknown, most of the species detected in our work can be found in other human niches. Work describing the oral fungal microbiome have reported that *Candida* species (mainly *C*. *albicans*, and others like *C*. *parapsilosis*) are the most frequent in the oral cavity, but also others like *Cladosporium*, *Aureobasidium*, *Saccharomycetales (S*. *cerevisiae* among others), *Aspergillus*, *Fusarium*, *Cryptococcus or Penicillium* ^24–27^. *Malassezia* has been described as the most common genus on human skin, but other genera like *Candida*, *Aspergillus* or *Penicillium* are also common inhabitants in this body niche^28^. Some fungal species found in our samples have also been detected in the human gut, namely *Candida* species, *Malassezia*, *Cladosporium*, *Debaromyces* and *S*. *cerevisiae* ^29,30^.

As some of the species found in our milk samples can also be detected in the oral cavity and the human skin, we cannot discard the possibility of transfer of fungi from the skin surrounding the breast, to the breastmilk, as well as from the baby’s skin or mouth transferred during suckling^31,32^. Another possible explanation would be that these fungal species originate from the mother’s mucosal surfaces, through an internal route, like the previously proposed entero-mammary pathway to explain the presence of bacteria in the mammary gland^33^. If this entero-mammary pathway exists, the presence of fungi in breastmilk could be more than the outcome of environmental contamination, and future work should aim to understand how fungal organisms reach human milk and if this pathway exists for fungi as well as bacteria. Whatever their origin, fungi are clearly present in breastmilk and are likely contributing to the establishment of infant microbiomes.

Culture techniques provide a complementary vision of fungal populations in breastmilk. Contrary to pyrosequencing results, the most abundant viable fungus detected was *Rhodotorula mucilaginosa*, which is a common airborne and highly ubiquitous fungus. It can also be found in the human body, where it can live as an opportunistic pathogen, particularly in immunocompromised patients^34,35^. The *Rhodotorula* genus is also part of the normal skin microbiota^28^. In the current study, we also found *Rhodotorula* DNA by pyrosequencing of breastmilk, but we could not classify any sequences to the species level even after quality filtering of the BLAST output. *Candida parapsilosis* was the second most prevalent strain isolated by culture techniques. Previous work has described this species as a normal commensal in the infant oral cavity^34,35^, and its presence has been reported in low birthweight infant’s stool^36^. Higher presence of this species in the infant gut has been correlated with higher risk of inflammatory bowel’s disease (IBD)^37^. *C*. *parapsilosis* sequences were also obtained in this work by high-throughput sequencing technology, although it was not among the most prevalent species.

The phenotypical and molecular characterization of the most prevalent isolates from our milk samples (*R*.*mucilaginosa and C*.*parapsilosis*) as well as the single *S*. *cerevisiae* isolate provided additional biological information regarding these organisms. The growth of all isolates at 37 °C and 42 °C demonstrated the ability of these strains to survive at human body temperature. However, none of them were able to grow in the presence of lactose as unique carbon source, which leads us to hypothesize that there are factors in breastmilk that allow their survival at higher temperatures and, further, that these species are utilizing alternative carbon sources in breastmilk.

The presence of an isolate highly similar to a commercial baker *Saccharomyces cerevisiae* strain (“Cinta roja”) in one of our samples is interesting. *S*. *cerevisiae* has traditionally been used in fermentative processes to produce beer, bread and wine, and is even consumed as a nutritional supplement, and humans can get in contact with it through diet and through its manipulation during baking. This strain could be a saprophytic colonizer of the human body as *S*. *cerevisiae* has been isolated from the digestive tract, vagina, skin and oropharynx of healthy hosts where their presence is benign and asymptomatic^38^. A recent study reported a decrease in *S*. *cerevisiae* levels in patients suffering from inflammatory bowel disease, whereas *Candida albicans* levels were higher compared to healthy patients^39^. *S*. *cerevisiae* var. *boulardii* is a yeast that has been used as dietary supplement due to its apparent probiotic effects^40,41^. Administration of *S*. *boulardii* has been shown to be associated with beneficial health effects in children with gastrointestinal disorders^42,43^. It also improves feeding tolerance and its supplementation in formula has shown beneficial effects on premature infants. However, some *S*. *cerevisiae* isolates have been responsible for human infections that are not life threatening except in severely immunocompromised patients where systemic infection may occur^22^. Pathogenic *Saccharomyces* clinical strains have shown resistance to high oxidative stress conditions^44^. Upon exposing the *S*.*cerevisiae* isolate to high hydrogen peroxide (H_2_O_2_) concentrations, in order to study its oxidative stress resistance, no differences were found when comparing with the other strains and the industrial *S*. *cerevisiae* T73 and “Cinta Roja” strains. The presence of H_2_O_2_ in breastmilk has an antibacterial role and when combined with lactoperoxidase (a milk peroxidase) and iodide, a strong anti-bacterial system is produced: the Lactoperoxidase system^45^. However, all of our tested isolates were able to survive under these conditions.

The discrepancies observed between culture isolation and culture-independent high throughput sequencing for the identification of fungi are not surprising. As we are currently unable to cultivate many microbial species under laboratory conditions, many fungal species may not be detected by standard culturing methods. In addition, fungi are present at much lower numbers, as compared to bacteria, in most human samples, making their isolation more difficult. Especially intriguing is the absence of *Malasezzia* isolates from our cultures, despite their high prevalence in pyrosequencing data. It remains to be determined whether the *Malasezzia* reads correspond to non-viable microorganisms. Future studies are needed to understand how breastmilk affects the viability of bacteria and fungi as well as the origin of fungi, such as *Malasezzia*, that are found in breastmilk.

*Malasezzia* yeasts are considered to be part of the normal microbiota of healthy individuals^28^. However, under some circumstances, they may act as opportunistic pathogens. Owing to their lipophilic nature, they colonize the seborrheic parts of the skin and they sustain themselves by using the fatty acids present in normal sebum, as they have the property of using lipids as a source of carbon^46^. This could favor their survival and growth in breastmilk, which contains high fat levels, but our results comparing breastmilk fungi and macronutrients did not show a positive correlation between the *Malassezia* genus and fat, as might be expected. We observed a positive correlation between the *Malassezia genus* and lactose, although this genus has been found to be unable to metabolize lactose^47^. In this study, *Malasezzia* was not isolated from any of the tested human milk samples and lactose utilization could not be assessed to confirm the correlation found. In the future, it will be important to establish the metabolic capabilities of *Malassezia* isolated from breastmilk with respect to sugars, in order to understand the observed correlation with lactose utilization.

Although we found positive relationships between fungal load and milk fat and non-fatty solids, no correlation was found with human somatic cells counts. Given that the number of somatic cells in milk is considered the gold standard for detecting bacterial infections (e.g. mastitis) in farm animals^48^, the absence of a somatic cell increase in our samples suggests a lack of significant immune response (although it cannot be ruled out that breastmilk fungi may be interacting with immune cells without causing inflammation). Thus, the data presented in the current work suggest that the presence of fungi in milk is not associated with infection in these healthy mothers without lactation problems. In addition, we find a highly significant negative correlation between bacterial load and human somatic cells, confirming that the presence of microorganisms did not correlate with an increase in somatic cells. This negative correlation, although not significant, was also found in a previous study with a lower number of samples^21^, confirming that this general measure (total somatic cell number) may not be a good method for detecting microbial infections in human breastmilk and that specific immune cells, such as polymorphonuclear leukocytes, could be more informative^49^.

The potential for fungi to enter the gastrointestinal tract via breastmilk should be considered as a mechanism for their initial colonization of the infant gut. In humans, the fungal composition of breastmilk has not been reported in healthy women and there are relatively few studies of fungal colonization patterns in the infant gut^39^. However, the impact of the fungal component of the gut microbiome could be relevant, and scarce information is available regarding gut bacteria-fungi relationships. Available data suggest a potential beneficial role of various fungi for human health^40–43^ and further work should be performed in order to better understand their mode of action.

## Conclusions

Through multiple methodological approaches, we have detected, isolated and identified fungi from human breastmilk. Although the natural presence of fungi in the milk of other mammals is well-accepted, there are no previous descriptions of specific fungal species in human samples from healthy mothers. Although their origin and role still remain to be elucidated, we show that fungi are clearly present in human breastmilk. Further research with larger cohorts should be performed in order to uncover their possible contribution to gut microbiota development and their potential role for infant health.

## Material and Methods

### Subjects and Sampling

Breastmilk samples from healthy lactating mothers (n = 65) within 1 month after birth were analyzed in this study (colostrum: n = 16; transitional milk: n = 14; mature milk samples: n = 28). Details of mode of delivery and gestational age were collected after birth. All infants were in good health.

Previous to sample collection, nipples and mammary areola were cleaned with soap and sterile water and soaked in chlorhexidine to reduce sampling of microorganisms residing on the skin. Milk samples were collected in a sterile tube manually, discarding the first drops. All samples were frozen at −20 °C until further processing.

Before sample collection, the mothers received oral and written information, and gave written informed consent to the protocol, which had been approved by the Ethics Committee of the Hospital Clínico Universitario de Valencia (Spain), and the Bioethics Subcommittee of Consejo Superior de Investigaciones Científicas (CSIC). All the methods were carried out in accordance with the relevant guidelines and regulations.

### Culture and Identification of Fungal Colonies

1 ml of each breastmilk sample was centrifuged 10 minutes at 9,000 rpm; fat was removed and pellets were resuspended in 400 μl of sterile water. 100 μl were plated in four solid fungal-selective media: Sabouraud (40 g/l dextrose, 10 g/l peptone and 20 g/l agar) supplemented with cloramphenicol 0.05 g/l (Roche); Rose Bengal (Conda-Pronadisa); CHROMagar^TM^ *Malassezia* and YPD (40 g/l dextrose, 40 g/l peptone, 20 g/l yeast extract and 40 g/l agar) supplemented with 25 μg/ml of streptomycin and 25 U/ml of penicillin (Biowest), and incubated aerobically at 37 °C. Positive control for Malasezzia CHROMagar medium was *Malassezia cuniculi* (CECT 13051; CBS 11721). Negative controls were included for each culture medium.

All isolated colonies were analyzed under the microscope to confirm fungal morphology and were further isolated to obtain single-cell pure colonies. DNA extraction was performed following the method described in detail in the Fungal DNA Isolation Section, and 4 μl were amplified by PCR using primers targeting the 18 S rRNA gene (forward: 5′-GTAGTCATATGCTTGTCTC; and reverse: 5′-CCATTCCCCGTTACCCGTTG); and the ribosomal Internal Transcribed Spacer (ITS) region, using ITS1F: 5′-GCTGCAACCATGGACTGGAT^50^; and 5.8 R: 5′-TCRATGGTGAAGTCAACGTG^51^ primers. PCR products were sequenced in an Applied Biosystems® 3730/3730xl DNA Analyzer at University of Valencia (Spain) and fungal isolates were identified by using the BLAST algorithm in the NCBI database, with minimum 98% sequence identity.

To test if all *C*. *parapsilosis* isolates and all *R*. *mucilaginosa* isolates, the two more prevalent isolates detected in our samples, were genetically identical, we performed a multiple alignment and generated a homology tree using DNAMAN software (version 7.212, Lynnon Corp., Canada).

### Phenotypic Characterisation of Fungal Isolates

#### Sugars Utilization and Growth at Different Temperatures

Isolates were plated on Yeast Nitrogen Base agar without aminoacids (Difco). Four different plates types were prepared: YNB without sugars, and YBN with a sole carbohydrate source (lactose, and glucose and sucrose as controls). Media were prepared by making a 10x concentrated stock solution with 6.8 g yeast nitrogen base powder, 1.5 g agar and 5 g of the selected sugar (except in the “no-sugar” plates), in 100 mL water, that was filtered and further diluted in 900 ml sterile water. Isolates were resuspended in 1 ml of sterile water and incubated at 30 °C degrees for 1 hour to induce starvation. 50 μl were plated in the corresponding medium and incubated at 30 °C until colonies appeared (1–3 days).

In order to check for temperature resistance, isolates were plated on GPY agar plates (0.5% w/v yeast extract (Pronadisa), 0.5% w/v peptone (Oxoid LTD), 4% w/v glucose (Panreac), and 2% w/v agar (Panreac)), and were incubated at three different temperatures: 28 °C, 37 °C and 42 °C until colonies appeared (1–3 days).

#### Resistance to Oxidative Stress

Strains were grown overnight in GPY agar plates at 30 °C. *S*. *cerevisiae* wine strain (T73) and baker strain (“Cinta Roja”) were included as controls. After adjusting to 0.1 OD in PBS, 6 mM hydrogen peroxide (H_2_O_2_) (Panreac) were added and samples were incubated for one hour at 30 °C with shaking. Dilutions 1:10, 1:100 and 1:1000 were done and 15 μl from each dilution were deposited in a drop on GPY agar plates. Plates were incubated for 48 h at 30 °C.

### Genetic Characterisation of Breastmilk *S*. *cerevisiae* Strain: mtDNA Restriction Patterns and δ-PCR Amplification Patterns Analysis

DNA of the *S*. *cerevisiae* FBMI18 isolate, as well as DNA from three *S*. *cerevisiae* control strains (Wine yeast T73, baker yeast “Cinta roja” and *S*. *boulardii*, which is a therapeutic *S*. *cerevisiae* strain marketed as Ultralevura® for probiotic purposes) were isolated according to De Llanos *et al*.^52^. The 5.8S-ITS region was amplified using the primers, PCR reaction conditions and thermal cycling parameters described previously by De Llanos *et al*.^53^.

The mtDNA restriction analysis was performed according to the method described by Querol *et al*.^54^. The amplified DNA (10 μl) was digested with Hinf I restriction endonuclease (Roche Molecular Biochemicals), following the supplier’s instructions. Restriction fragments were separated in 0.8% (w/v) agarose (Pronadisa) gels in 1 × TAE buffer. Electrophoresis gels were stained with ethidium bromide (0.5 µg/ml) (Sigma-Aldrich Chemie) and visualized with UV light. The DNA of phage λ digested with Pst I (Roche Molecular Biochemicals) served as size standard.

δ sequences were amplified in a GeneAmp PCR System 9700 (Perkin Elmer, California, USA) using the primers, PCR reaction conditions and thermal cycling parameters described previously by De Llanos *et al*.^53^. The PCR products were separated on 1.4% (w/v) agarose (Pronadisa) gel in 1 × TAE buffer. After that, electrophoresis gels were stained and visualised as described above.

### FISH Detection of Fungi in Milk

Fluorescent *in situ* hybridization (FISH) was performed on 5 milk samples to detect fungi, as an alternative method to DNA sequencing and culture-based methods, by using the Euk516 probe (5′-ACCAGACTTGCCCTCC) targeting the 18 S rRNA gene^55^, labeled with fluorescein isothiocyanate (FITC) at the 5′ end. Suspensions of previously fixed breastmilk samples (fixed with paraformaldehyde at 4% final concentration, overnight incubation) were vortexed thoroughly, and 100 μl aliquots were dispensed to new microcentrifuge tubes and pelleted (6000 rpm, 10 min). Supernatant was removed and cell pellets were resuspended in 100 μl of hybridization buffer preheated to 50 °C. Hybridization buffer consisted of 20 mM Tris [pH 8.0], 0.9 M NaCl, 0.01% SDS and miliQ water. One microliter of the probe was added to the mix (concentration 100 mM), and suspensions were hybridized at 53 °C overnight on an AccuBlock^TM^ heat block. After that, 500 μl wash buffer (hybridization buffer without probe, preheated to 50 °C) was added, samples were vortexed and centrifuged 5 min at 9,000 rpm and pellets resuspended in 100 μl of PBS. Calcofluor White Stain (Sigma-Aldrich) at 0.01% was added to the suspension and used as second marker, as it binds to cellulose and chitin of fungal cell walls^56^. An isolated strain of *Candida parapsilosis* from one of our samples was included as positive control (FBMI4). Samples were visualized with fluorescence microscopy using a Nikon Eclipse E90i microscope (Nikon Corporation) with a 100 × objective. Images were processed using NIS-Elements BR v3.22 software (Nikon).

### Fungal DNA Isolation

Milk samples (5 ml) were thawed and centrifuged at 4,000 x g for 20 minutes to separate fat and cells from whey. Thereafter, total DNA was isolated from the pellets by using the MasterPure Complete DNA & RNA Purification Kit (Epicentre) according to the manufacturer’s instructions with some modifications^57^. 250 μl of sterile saline solution and 250 μl of lysis buffer were added to the pellets, together with a mix of 150–212 μm and 425–600 μm, acid-washed glass beads (Sigma). To enhance the disruption of fungal cell walls, samples were put through three cycles of vigorous mixing in a TissueLyser II (QIAGEN) 5 min at 30 Hz, incubation in dry ice 3 minutes and 5 minutes at 65 °C in a heat block. Lysozyme (20 mg/ml) and zymolyase (0.25 mg/ml) were added to the tubes, and samples were incubated for 1 h at 37 °C. 2 μl of proteinase K were added and samples were incubated for 15 minutes at 65 °C. The reaction was stopped by putting tubes on ice and proteins were precipitated using 350 μl of MPC Protein Precipitation Solution, and discarding the pellets. DNA was precipitated using isopropanol, washed with 70% Ethanol and resuspended with 30 μl TE buffer. The total DNA isolated was quantified with a Qubit^TM^ 3 Fluorometer (ThermoScientific).

### Quantitative Real-time Polymerase Chain Reaction Analysis of Fungal Load

qPCR amplification and detection were performed with primers targeted to the conserved ITS1–5.8S rRNA fungal region, described in detail in the Culture and Identification of Fungal Colonies Section, using an annealing temperature of 61 °C in a Light Cycler 480 Real-Time PCR System (Roche Technologies). Each reaction mixture of 20 μl was composed of 10 μl of KAPA Sybr Fast qPCR Kit (KAPA Biosistems), 0.4 μl of each primer (10 μM concentration) and 2 μl of template DNA. All amplifications were performed in duplicates and a negative control was included in each qPCR reaction run. The fungal concentration in each sample was calculated by comparison with the Ct values obtained from a standard curve. These were generated using serial ten-fold dilutions of DNA extracted from 10 million fungal cells from 5 pure cultures from different fungal species (*Candida albicans*, *Malassezia cuniculi*, *Sacharomyces boulardii*, *Thrichosporon cutaneum* and *Mucor circinelloides*), that were pooled to create a single standard curve. Fungal cells were quantified and sorted using a BD FACSAriaTM II cytometer after mild sonication to separate aggregated cells. Under the described PCR conditions, the fungal primers did not amplify human or bacterial DNA, when using DNA from human umbilical vein endothelial cells (Advancell, Spain)^58^ and a mix of bacterial-species DNA (*Staphylococcus epidermidis*, *Pseudomonas aeruginosa*, *Streptococcus mitis*, *Bifidobacterium dentium* and *Rothia mucilaginosa*).

Averages were calculated for duplicates of Ct values in every sample, and inconsistent duplicates were re-run. A standard curve was included in each run (three runs were needed in total). Milk samples that showed Ct values equal or higher than the negative control were considered to be negative for fungal DNA. One-way ANOVA (Kruskal-Wallis test) was performed for groups comparison, using GraphPad PRISM^R^ 6 (GraphPad Software).

Bacterial load data from the same samples were also obtained using primers targeting the bacterial universal single-copy gene fusA^21^.

### PCR Amplification and Sequencing

Fungal DNA for sequencing was amplified by PCR from the 15 milk samples with the highest fungal load previously obtained by qPCR, using universal fungal primers against the 28S rRNA gene: LR0R: 5′-ACCCGCTGAACTTAAGC; and LR3: 5′ CCGTGTTTCAAGACGGG^59^, by the use of high-fidelity AB-Gene DNA polymerase (ThermoScientific) with an annealing temperature of 52 °C and 20 cycles. A secondary amplification was performed by using the purified PCR product as a template, in which the universal primers were modified to contain the pyrosequencing adaptors A and B and an 8-bp “barcode” specific to each sample, following the method used in Benitez-Paez *et al*.^60^. Negative controls were included in primary and secondary amplifications using water and the purified PCR product from the primary amplification, respectively. The purification of the 610 bp PCR products was performed with NucleoFast 96 PCR filter plates (Macherey-Nagel), and the final concentration of the DNA per sample was measured with a Qubit^TM^ 3 Fluorometer (ThermoScientific).

PCR products were pyrosequenced from the reverse primer end using a 454 Life Sciences system, in a GS-FLX sequencer with Titanium chemistry (Roche) at Macrogen Korea (Rep. of Korea). A negative control for the sequencing run was also included by Macrogen to discard contamination. The amplification of a long 28S rRNA region increases accuracy in taxonomic assignment due to the implementation of a machine-learning algorithm from the Ribosomal Database Project (RDP) platform^59^ and to the amplification of fragments with the same size, as opposed to ITS regions of variable length that would be differentially sequenced in second-generation sequencing platforms.

## Data Analysis

Sequences with an average quality value <20 and/or with >4 ambiguities in homopolymeric regions in the first 360 flows were excluded from the analysis. 28S rRNA gene reads were end-trimmed in 20 bp sliding windows with average quality value >20, then length (>400 bp) and quality filtered (average Q > 20), through the Galaxy server (http;//getgalaxy.org/). Chimeric reads were eliminated using UCHIME^61^, and only sequences longer than 400 bp were considered, resulting in a total of 17,000 reads with a mean of 1,700 ± 444.9 (SE) sequences per sample. Sequences were assigned to each sample by the 8-bp barcode and phylum-, family- and genus-level taxonomic assignment of sequences that passed quality control were made using the Ribosomal Database Project classifier software^62^ within an 80% confidence threshold. Sequences >97% identical were considered to correspond to the same operational taxonomical unit (OTU), representing a group of sequences that presumably correspond to the same species^30^. Sequences were clustered at 97% nucleotide identity over 90% sequence alignment length using the CD-hit software^63^.

BLASTn was performed against the RDP LSU database for taxonomically assigning reads at the species level^64^. Only top hits with >98% similarity and >400 bp alignment length were considered.

### Milk Macronutrient Composition Analysis

Fat, protein and lactose composition (% w/w) was analyzed by spectrophotometry using a MilkoScan FT 6000 (FOSS). Somatic cells (cells/ml) were determined using an Integrated Milk Testing Fossomatic FC(FOSS) cytometer, in LICOVAL-UPV, Polytechnic University of Valencia (Spain), when the samples’ volume was sufficient (n = 34).

### Statistical Analysis

R software (version 3.2.2)^65^ was used for computing Spearman’s correlation coefficients using the stats package, and heatmap plots with gplots package^66^. Other statistical analysis and graphs were performed using GraphPad PRISM^R^ 6 (GraphPad Software).

### Data Availability

The datasets generated during the current study are available in the European Nucleotide Archive (ENA, EMBL-EBI) repository at http://www.ebi.ac.uk/ena/data/view/PRJEB19310, with accession number PRJEB19310.

## Electronic supplementary material

Supplementary Figures
