# Author Correction: Multiple Approaches Detect the Presence of Fungi in Human Breastmilk Samples from Healthy Mothers

**DOI:** 10.1038/s41598-018-35165-1

**Published:** 2018-11-09

**Authors:** Alba Boix-Amorós, Cecilia Martinez-Costa, Amparo Querol, Maria Carmen Collado, Alex Mira

**Affiliations:** 1 Institute of Agrochemistry and Food Technology, Spanish National Research Council (IATA-CSIC), Department of Biotechnology, Av. Agustin Escardino 7, 46980 Valencia, Spain; 2 Department of Health and Genomics, Center for Advanced Research in Public Health, FISABIO Foundation, Valencia, Spain; 3 Department of Paediatrics. University of Valencia, Paediatric Gastroenterology and Nutrition Section, Hospital Clínico Universitario de Valencia (Spain), Blasco Ibáñez Av., 17, 46010 Valencia, Spain

Correction to: *Scientific Reports* 10.1038/s41598-017-13270-x, published online 12 October 2017

This Article contains an error in the Material and Methods section under the subheading ‘Culture and Identification of Fungal Colonies’,

“DNA extraction was performed following the method described in detail in the Fungal DNA Isolation Section, and 4 μl were amplified by PCR using primers targeting the 18S rRNA gene (forward: 5′-GTAGTCATATGCTTGTCTC; and reverse: 5′-CCATTCCCCGTTACCCGTTG); and the ribosomal Internal Transcribed Spacer (ITS) region, using ITS1F: 5′-GCTGCAACCATGGACTGGAT^50^; and 5.8 R: 5′-TCRATGGTGAAGTCAACGTG^51^ primers.”

should read:

“DNA extraction was performed following the method described in detail in the Fungal DNA Isolation Section, and 4 μl were amplified by PCR using primers targeting the 18S rRNA gene (forward: 5′-GTAGTCATATGCTTGTCTC; and reverse: 5′-CCATTCCCCGTTACCCGTTG); and the ribosomal Internal Transcribed Spacer (ITS) region, using ITS1F: 5′-TCCGTAGGTGAACCTGCGG^50^; and 5.8 R: 5′-CGCTGCGTTCTTCATCG^51^ primers.”

