# Supplementary material for: Multiple Approaches Detect the Presence of Fungi in Human Breastmilk Samples from Healthy Mothers
Source: Sci Rep. 2017 Oct 12;7:13016. doi: 10.1038/s41598-017-13270-x (PMC5638952; doi:10.1038/s41598-017-13270-x)
Supplement: Supplementary file 1 — Supplementary Figures [file 41598_2017_13270_MOESM1_ESM.pdf]

# Multiple approaches detect the presence of fungi in human breast milk samples from healthy mothers.

Alba Boix-Amorós, Cecilia Martinez-Costa, Amparo Querol, Maria Carmen Collado*, Alex Mira*.

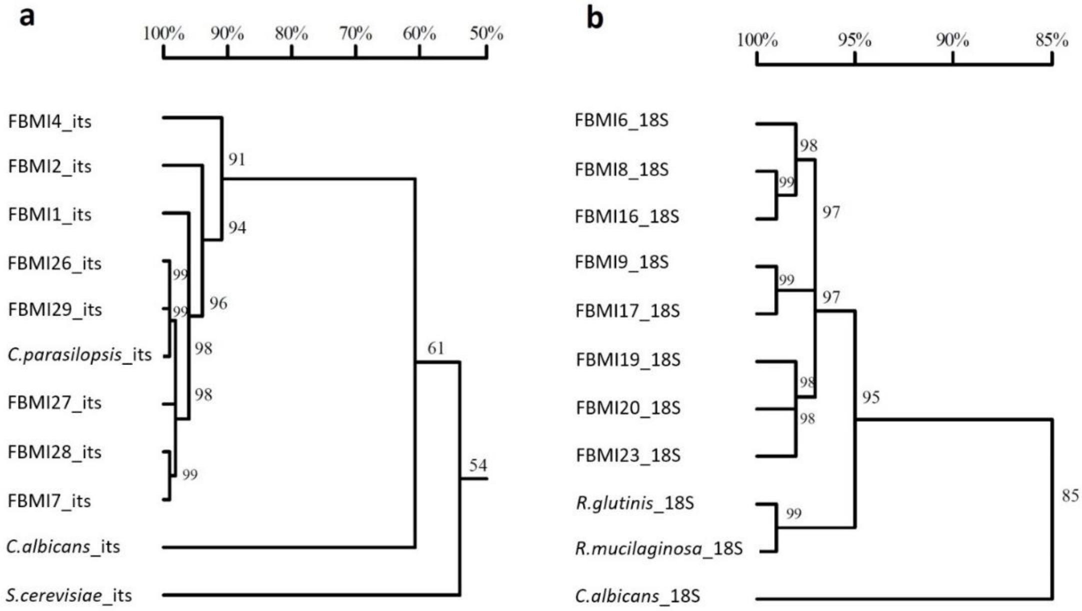


**Supplementary Figure 1. Homology trees for *C.parapsilosis* and *R.mucilaginosa* isolates from breast milk. (a)** Shows a homology tree for *Candida parapsilosis* isolates from breast milk samples (n=8) PCR-amplified with ITS1-5.8 primers and Sanger-sequenced. Three reference strains were included as controls (*C.parapsilosis* , *C.albicans* and *S.cerevisiae*). **(b)** Shows a homology tree for *Rhodotorula mucilaginosa* isolates from breast milk samples (n=8) PCR-amplified with 18S rRNA primers and Sanger-sequenced. Three reference strains were included as controls (*R.mucilaginosa, R.glutinis* and *C.albicans*). Multiple alignments and homology trees were performed with DNAMAN software.

**a**

| Strains | 28°C | 37°C | 42°C | No sugar | Lactose | Glucose | Sucrose |
|---|---|---|---|---|---|---|---|
| FBMI6 (R.mucilaginosa) | ++ | ++ | + | - | - | ++ | ++ |
| FBMI7 (C.parapsilosis) | ++ | ++ | ++ | - | - | ++ | ++ |
| FBMI18 (S.cerevisiae) | ++ | ++ | ++ | - | - | ++ | ++ |
| Cinta Roja | ++ | ++ | ++ | - | - | ++ | ++ |
| S.cerevisiae T73 | ++ | - | - | - | - | ++ | ++ |

**b**

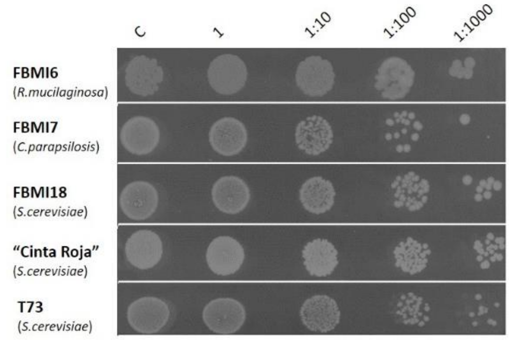


**c**

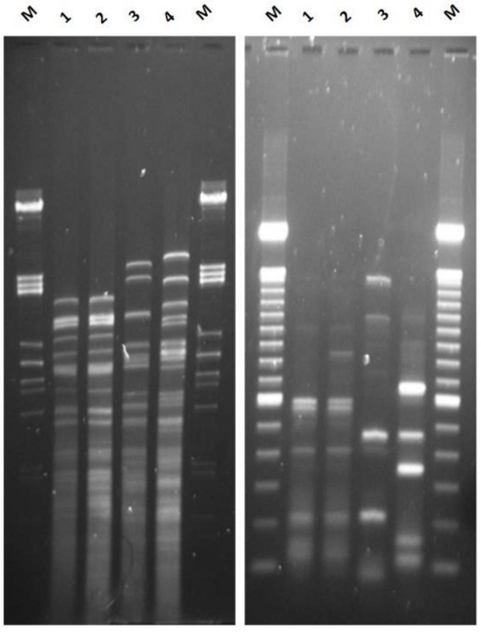


**Supplementary Figure 2. Phenotipic and genetic characterization of fungal isolates from human breastmilk. a) Isolates viability at different temperatures and utilization of different sugars.** (-) indicates no growth, (+) indicates moderate growth, and (++) indicates high growth. **b) Isolates' resistance to oxidative stress.** The picture shows the differential growth of isolates after 1-hour exposure to 6mM $H_20_2$. The first column corresponds to the growth of isolates without $H_20_2$ exposure. The remaining columns of growth correspond to isolates exposed to $H_20_2$: 1(no dilution), and dilutions 1:10, 1:100 and 1:1000. **c) Hinf I mtDNA restriction patterns (HinfI) and δ-PCR amplification patterns (δ) of the DNA of yeast strains**. 1 (FBMI18 strain), 2 (Baker's yeast "Cinta roja"), 3 (Wine strain T73), 4 (*S.boulardii*, Ultralevura). The DNA of phage λ digested with Pst I (Roche Molecular Biochemicals) and a 100-bp DNA ladder marker (Gibco BRL, Gaithersburg, MD.) served as the size standard respectively (M).
